# A cross-sectional study on performance evaluation in Italian standardbred horses’ real-time PCR-positive for Theileria equi

**DOI:** 10.1186/s12917-024-03908-0

**Published:** 2024-03-05

**Authors:** Pierpaolo Coluccia, Manuela Gizzarelli, Maria Teresa Scicluna, Giuseppe Manna, Valentina Foglia Manzillo, Francesco Buono, Luigi Auletta, Veronica Palumbo, Maria Pia Pasolini

**Affiliations:** 1 Interdepartmental Center of Veterinary Radiology, University of Napoli Federico II, Via Federico Delpino 1, Napoli, 80137 Italy; 2 Department of Veterinary Medicine and Animal Productions, University of Naples Federico II, Via Federico Delpino 1, Naples, 80137 Italy; 3 Istituto Zooprofilattico Sperimentale del Lazio e della Toscana “M. Aleandri”, Via Appia Nuova 1411, Rome, 00178 Italy; 4 Department of Veterinary Medicine and Animal Sciences (DIVAS), University of Milano, via dell’Università 6, Lodi, 26900 Italy

**Keywords:** Italian Standardbred horses, Performances, Theileria equi, Chronic equine piroplasmosis, Real-time PCR, Haematology

## Abstract

**Background:**

Inflammatory myopathy and perivasculitis have been recently described in horses with chronic equine piroplasmosis (EP). These alterations may be linked to poor performances. The aims of this study were to evaluate the prevalence for EP in clinically healthy Italian Standardbred (IS) racehorses and to compare laboratory parameters and performance metrics between positive and negative horses. Real-time PCR was applied for the detection of *T. equi* and *B. caballi* positivity. Haematology parameters, blood chemistry results, subjective muscle mass scores, and performance metrics were compared between PCR-positive and -negative horses.

**Results:**

This cross-sectional study included 120 well-trained IS racehorses and was performed over a two-years period. The prevalence of *T. equi* was 36.3%, whereas all samples were negative for *B. caballi*. Red blood cells count, haemoglobin concentration, aspartate aminotransferase, alkaline phosphatase, and gamma-glutamyl transferase activities were significantly higher in PCR-positive horses, whereas blood urea nitrogen, globulin concentration and globulin-to-albumin ratio were significantly lower in PCR-positive horses compared to PCR-negative ones. Nonetheless, all values fell within the physiological range. The best racing time, which was selected as the most representative of the performance metrics at the principal component analysis, was not affected by PCR positivity, the muscle mass score or the training yard. The best racing time was significantly better in horses with a mild or no signs of muscular atrophy, within the PCR-positive group. The muscle mass score was associated with the training yard in PCR-negative horses.

**Conclusions:**

Prevalence of *T. equi* was high in IS racehorses in southern Italy. The absence of obvious changes in haematological and biochemical parameters, as well as performance metrics in positive horses, highlights the need for specific diagnostic tests to identify chronically infected horses.

## Background

Equine piroplasmosis (EP) is an infectious, tick-borne disease caused by the intraerythrocytic protozoans *Theileria equi* and *Babesia caballi* that affect equids, which are intermediate hosts. High rates of seropositivity have been reported in Italy [1–5]. Seropositive racehorses can exhibit decreased performance, and stress due to intense athletic activity can lead horses to develop clinical manifestations of the disease [6, 7]. However, there are few data concerning the relationship between poor performance syndrome and EP in racehorses, and no report has been published regarding Italian Standardbreds (IS) [8–10].

The failure of horses to meet performance expectations is considered poor performance, and myopathies may be a potential cause [11]. Inflammatory myopathy and perivasculitis have been recently described in horses with chronic EP, as muscle damage results from the development of autoantibodies against muscle antigens [9].

The aims of the present study were: • to evaluate the prevalence of PCR positivity for *T. equi* and *B. caballi* in clinically healthy IS racehorses; • to compare haematological and biochemical values, and performances between PCR-positive and PCR-negative horses.

Our hypotheses were as follows: • PCR positivity is common among clinically healthy sport IS horses; • the haematological and serum biochemical parameters, and performances of clinically healthy PCR-positive and PCR-negative IS racehorses do not differ.

## Results

### PCR results

**Fig. 1:**
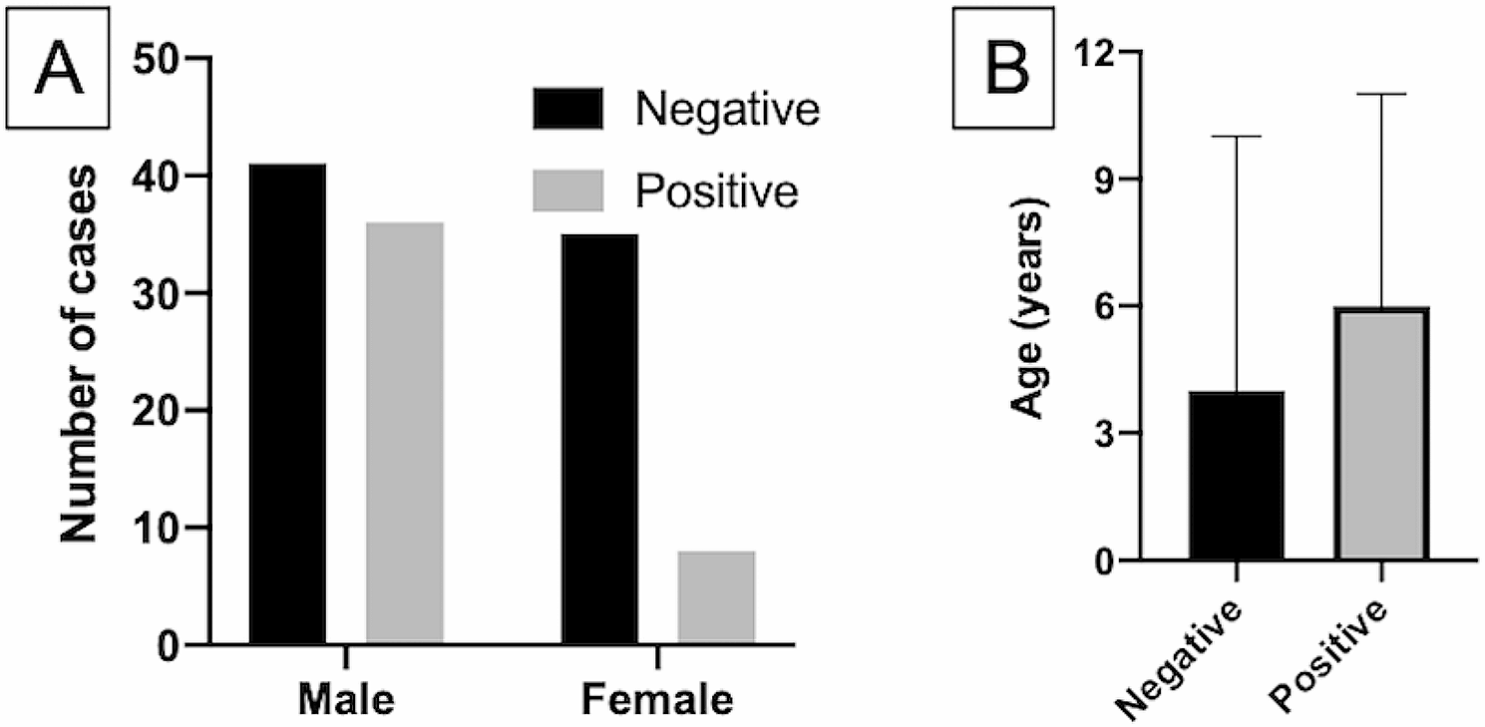
(**a**) Number of PCR-negative and -positive horses by sex. (**b**) Median ages of negative and positive horses. Bars indicate maximum values

A sample of 130 horses would have ensured 80% power to detect a difference between the positive and negative groups with two-sided test at *P* = 0.05. The exclusion criteria led to a final sample of 120 IS, aged 3 to 11 years, including 77 males and 43 females. Thus, the analysis resulted in a power of 74%. All ISs were PCR negative for *B. caballi*; 76 horses (63.3%) were PCR-negative, and 44 (36.7%) horses were PCR-positive for *T. equi*. Forty-one males (53.25%) were negative, and 36 males (46.75%) were positive, whereas 34 (81.40%) females were negative, and 8 females (18.60%) were positive. Males and females significantly differed regarding PCR positivity/negativity (*P* = 0.003, Fig. 1A), with females appearing to be more protected from infection than males (O.R.=0.26, 95%CI 0.11–0.64; R.R.= 0.65, 95%CI 0.50–0.85). PCR-negative horses had a median age of 4 years (3–10 years), and PCR-positive horses had a median age of 6 years (3–11 years); positive horses were significantly older (*P* = 0.008, Fig. 1B).

None of the horses enrolled had an history of severe bodyweight loss or muscle atrophy nor of haematological alterations compatible with EP, as reported by the owners. Management practices, including both training schedule and foraging habits, were considered superimposable at the evaluation of the clinician (M.P.P.). No statistical evaluation was performed on the aforementioned anamnestic and management variables.

On the overall sample, mean bodyweight was 445 ± 40 kg; mean bodyweight in the PCR-positive group was 444 ± 38 kg, and it was 446 ± 42 kg in the PCR-negative group, with no difference between the two groups (*P* = 0.80).

### Haematology and serum biochemistry results

All results for the complete blood counts (CBC) are summarized in Table 1. Mean and median values of all CBCs and chemistry parameters were within the normal physiological range for horses according to the laboratory reference values. In PCR-negative horses, the number of RBCs (8.77 ± 0.65 × 10^3^/µL), the Hgb concentration (15.4 ± 1.9 gr/dL), and the MCHC (39.8; 33.6–47.9) were significantly higher than those in PCR-positive horses (8.47 ± 0.78 × 10^3^/µL; 14.5 ± 2.3 gr/dL; and 35.4–33.7–45.8 g/dl; *P* = 0.02, *P* = 0.01, and *P* = 0.02, respectively).

**Table 1:** Complete blood count in the two groups. Data are reported as mean (SD) or median (range), according to distribution

	Negative	Positive	Statistic	*P* value
**RBC** (x 10^6^/µL)	8.8 (0.6)	8.5 (0.8)	t = 2.3, d.f.=118	0.02*
**Hgb** (g/dL)	15.4 (1.9)	14.5 (2.3)	t = 2.5, d.f = 118	0.01*
**Hct** (%)	39.7 (3.2)	38.8 (4.5)	t = 1.2, d.f.=69	0.23
**MCV** (fL)	45.4 (2.6)	46.0 (39.4–51.0)	U = 1432	0.19
**MCH** (pg)	17.6 (1.6)	17.1 (2.0)	t = 1.6, d.f.=118	0.12
**MCHC** (g/dL)	39.8 (33.6–47.9)	35.4 (33.7–45.8)	U = 1254	0.02*
**RDW** (%)	17.8 (0.6)	17.6 (16.4–24.1)	U = 1400	0.14
**Plt** (x 10^3^/µL)	149.7 (32.4)	140.6 (44.6)	t = 1.2, d.f.=69	0.24
**Pct** (%)	0.12 (0.05–0.45)	0.12 (0.06–0.40)	U = 1617	0.76
**PDW** (%)	10.1 (9.6–11.8)	10.1 (9.5–11.9)	U = 1619	0.77
**WBC** (x 10^3^/µL)	8.2 (1.4)	8.3 (1.4)	t = 0.5, d.f.=118	0.64
**Lym** (%)	20.0 (13.0–67.0)	19.0 (15.0–31.0)	U = 1508	0.43
**Neu** (%)	69.0 (19.0–82.0)	70.0 (50.0–79.0)	U = 1468	0.31
**Mon** (%)	4.0 (0.0–10.0)	3.0 (0.0–13.0)	U = 1367	0.11
**Eos** (%)	6.0 (1.0–10.0)	5.5 (2.0)	U = 1561	0.62
**Bas** (%)	0.0 (0.0–6.0)	0.0 (0.0–1.0)	U = 1631	0.93

* *P* < 0.05. t represents both Student’s t test or Welch’s correction; d.f.: degree of freedom; U represents Mann-Whitney’s U test. Abbreviations: Basophils (Bas); Eosinophils (Eos); Hematocrit (Hct); Hemoglobin (Hgb); Lymphocytes (Lym); Mean Cell Hemoglobin Concentration (MCHC); Mean Cell Volume (MCV); Mean Corpuscular Hemoglobin (MCH); Monocytes (Mon); Neutrophils (Neu); Plateletcrit (Pct); Platelet Distribution Width (PDW); Platelets (Plt); Red Blood Cells (RBC); Red cell Distribution Width (RDW); White Blood Cells (WBC)

All results of serum biochemistry are summarized in Table 2. In PCR-negative horses, the Glob concentration and the Alb/Glob ratio were significantly lower compared to those in PCR-positives horses (*P* = 0.049 and *P* = 0.03, respectively). The BUN value was significantly increased in PCR-positive horses compared to PCR-negative horses (*P* = 0.005). Serum AST, AlkP, and GGT values were significantly higher in PCR-negative horses than in PCR-positive horses (*P* = 0.02, *P* < 0.0001, and *P* = 0.02, respectively).

**Table 2:** Serum biochemistry in the two groups. Data are reported as mean (SD) or median (range), according to distribution

	Negative	Positive	Statistic	*P* value
**BUN** (mg/dL)	14.8 (3.2)	17.0 (10.0–27.0)	U = 1063	0.005**
**TBil** (mg/dL)	2.5 (0.7)	2.5 (1.5–5.4)	U = 1324	0.42
**Crea** (mg/dL)	1.3 (0.8–2.0)	1.3 (1.0–1.8)	U = 1421	0.81
**BUN/Crea**	11.0 (6.0–18.0)	12.2 (2.7)	U = 1112	0.09
**Glu** (mg/dL)	84.0 (52.0–140.0)	85.0 (56.0–140.0)	U = 1327	0.49
**TP** (g/dL)	6.5 (4.4–8.6)	6.5 (6.1–8.8)	U = 1306	0.30
**Alb** (g/dL)	2.8 (1.8–3.4)	2.8 (2.5–3.6)	U = 1272	0.18
**Glob** (g/dL)	3.6 (2.6–5.2)	3.8 (2.7–5.5)	U = 1118	0.049*
**A/G**	0.8 (0.6–1.0)	0.7 (0.6–1.3)	U = 1105	0.03*
**Ca** (mg/dL)	11.9 (8.2–16.0)	11.8 (10.9–15.3)	U = 1367	0.58
**AST** (U/L)	372 (211–1083)	343 (171–1083)	U = 1107	0.02*
**AlkP** (U/L)	142.1 (34.8)	114.5 (25.6)	t = 4.78, d.f.=99	< 0.0001**
**GGT** (U/L)	26.0 (9.0–98.0)	22.0 (5.0–109.0)	U = 1080	0.02*
**CK** (U/L)	117.0 (10.0–1885.0)	116.5 (68.0–215.0)	U = 1280	0.23
**LDH** (U/L)	731.0 (345.0–1823.0)	729.9 (258.6)	U = 1358	0.47

* *P* < 0.05; ** *P* < 0.01. t represents both Student’s t test or Welch’s correction; d.f.: degree of freedom; U represents Mann-Whitney’s U test. Abbreviations: Albumin (Alb); Alkaline Phosphatase (AlkP); Aspartate amino-transferase (AST); Blood Urea Nitrogen (BUN); Calcium (Ca); Creatinine (Crea); Creatin-Kinase (CK); Gamma Glutamyl-Transferase (GGT); Glycemia (Glu); Globulin (Glob); Lactate Dehydrogenase (LDH); Total Bilirubin (TBil); Total Proteins (TP)

**Fig. 2:**
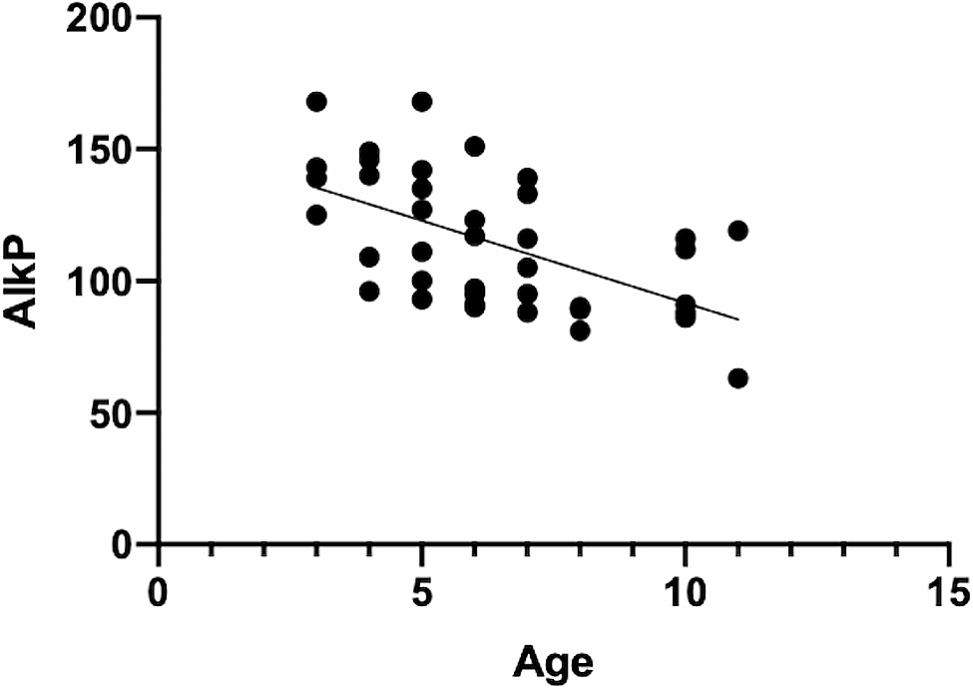
Graphic representation of the negative correlation between serum alkaline phosphatase (AlkP) activity and age (in years). The line represents the regression line

Within-groups analyses did not indicate any effects of signalment data on RBC count, Hgb, BUN, Alb/Glob, or AST values in either the PCR-positive or PCR-negative group. In the PCR-positive group, bodyweight affected the MCHC, and age affected the AlkP concentration (Table 3), whereas in the PCR-negative group, age affected the Glob concentration (Table 3). Although a significant effect was detected in the general linear model, no significant correlation between bodyweight and the MCHC (r_s_=-0.112, *P* = 0.48) or between age and Glob concentration (r_s_=0.173, *P* = 0.14) was detected, and no trend could be visually identified. A significant negative correlation between age and AlkP (r_s_=-0.609, *P* < 0.0001) was detected in the PCR-positive group (Fig. 2).

**Table 3:** Univariate general linear model for within groups signalment data effects on variables significantly different between groups

	Positive group				Negative group			
Effect	Type III q. sum	d.f.	F	*P*	Type III q. sum	d.f.	F	*P*
MCHC								
Bodyweight	542.700	37	41.907	0.005**	661.616	53	1.646	0.153
Glob								
Age	2.043	7	1.467	0.217	3.446	6	4.027	0.017*
AlkP								
Age	9,624.686	7	2.768	0.024*	5,404.437	6	1.217	0.358

* *P* < 0.05; ** *P* < 0.01. q.: quadratic; d.f.: degree of freedom; F represents statistic. Abbreviations: Alkaline Phosphatase (AlkP); Globulin (Glob); Mean Cell Hemoglobin Concentration (MCHC)

### Muscle atrophy

**Fig. 3:**
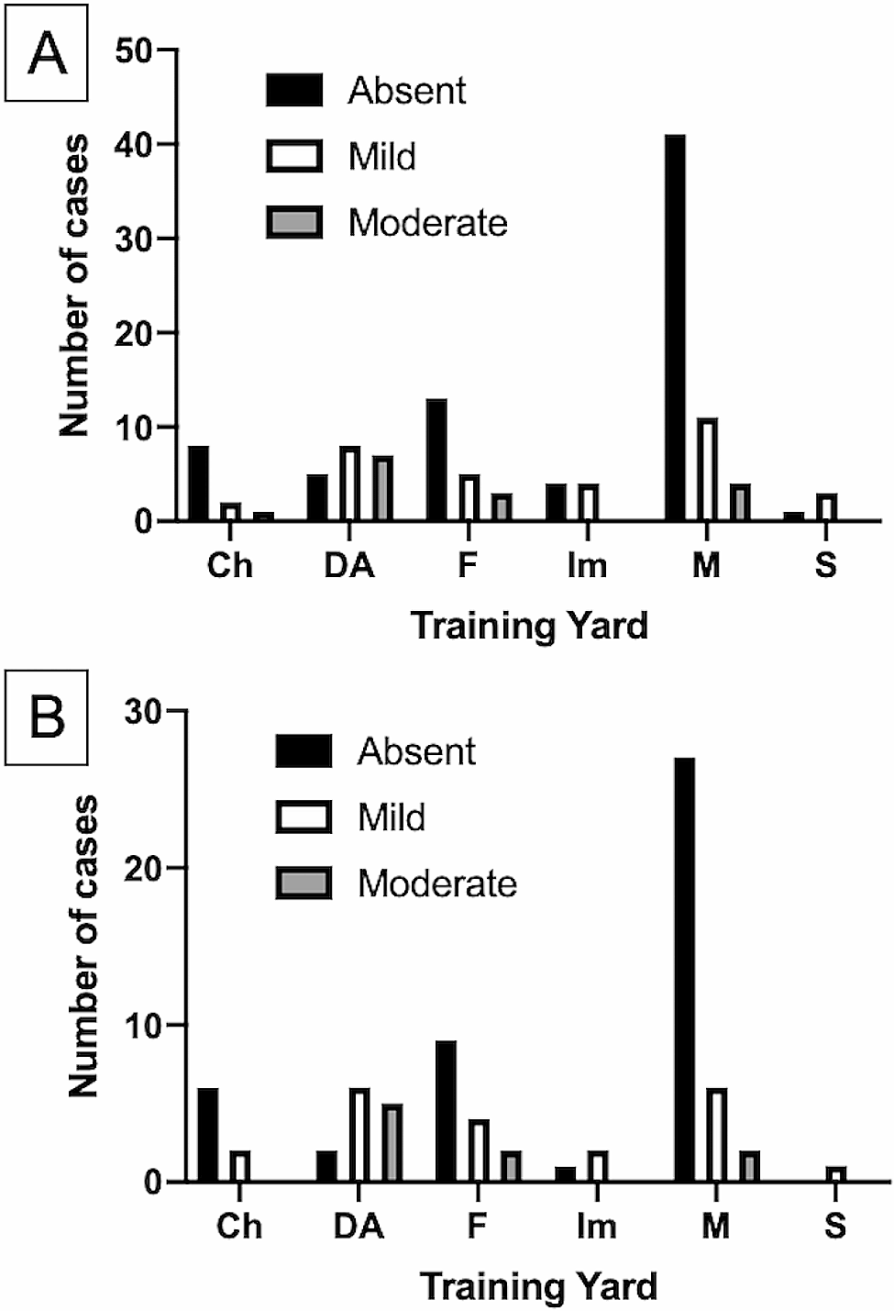
Graphic representation of the distribution of the MMS in the various training yards (**a**) in the whole sample and (**b**) within the PCR-negative group. In black, MMS = 0; in white, MMS = 1; in grey, MMS = 2

Among the overall sample, thirty-three (27.5%) horses showed a mild degree (MMS of 1) of muscular atrophy, fifteen (12.5%) horses showed a moderate degree (MMS of 2) of muscular atrophy, and 72 (60%) horses showed no signs of muscular atrophy (MMS of 0); none of the horses enrolled exhibited a severe degree (MMS of 3) of muscular atrophy. The MMS was not associated with PCR positivity (*P* = 0.95), whereas it was associated with the training yard (*P* = 0.004, Fig. 3A), in the overall sample; such association was confirmed within the PCR-negative group (*P* = 0.008, Fig. 3B), but not within the PCR-positive group (*P* = 0.55).

### Performance metrics

All performance metrics resulted highly correlated between each other (*r* > 0.8, *P* < 0.0001). The PCA analysis performed on the original performance metrics, i.e., the number of total and placed races, number of victories, mean racing speed, the best racing time and total earnings, showed that the best racing time explained alone 55.6% of the variance with no need to introduce other explaining factors (*P* < 0.0001). The best racing time was not affected by PCR positivity, the MMS or the training yard. This result was confirmed in the PCR-negative group, in which the best racing time did not differ by the MMS, the training yard or the MMS within the training yard. In the PCR-positive group, the best racing time was significantly better in horses with a lower MMS, i.e., with mild or no detectable atrophy, but it was not affected by the training yard or by the MMS within the training yard. Median number of races did not differ between PCR-positive and PCR-negative horses (30, 7–30; 30, 4–30; *P* = 0.30).

**Fig. 4:**
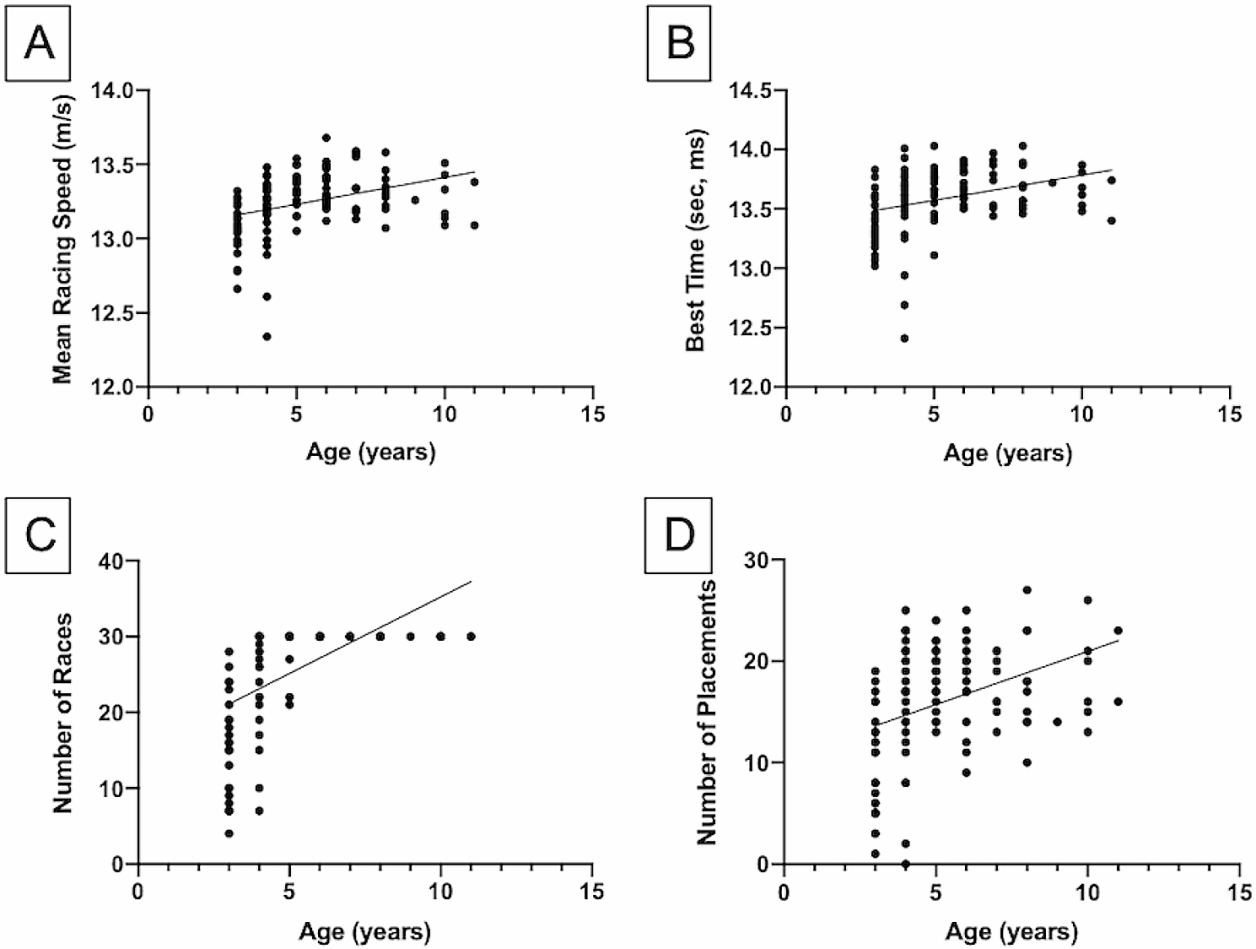
Graphic representation of the correlations between signalment data and performance parameters in the whole sample. The line represents the regression line. (**a**) Correlation between the mean racing speed (in m/s) and age (in years). (**b**) Correlation between the best time (in s, ms) and age (in years). For the sake of simplicity, minutes have been excluded from the best time, which should be considered to be with 01 min (e.g., 01 min 13 s 8 milliseconds). (**c**) Correlation between the number of races and age (in years). (**d**) Correlation between the number of placements and age (in years)

Among the signalment data, age of the horse significantly influenced the mean racing speed, the best racing time, the number of races ran and the number of placements. *Post hoc* analysis confirmed all the significant correlations: mean racing speed and age, with older horses having a faster mean speed (r_s_=0.467, *P* < 0.0001; Fig. 4A); best racing time and age, with younger horses displaying better times (r_s_=0.404, *P* < 0.0001; Fig. 4B); and numbers of races and placements and age, with older horses having participated in more competitions (r_s_=0.764, *P* < 0.0001; Fig. 4C) and having obtained more placements (r_s_=0.400, *P* < 0.0001; Fig. 4D), respectively. Significant results are reported in Table 4. Such correlations were confirmed both in the PCR-positive and PCR-negative groups.

**Table 4:** Results of the univariate general linear model for significant effect of signalment on performances, in the whole sample

Performance	Effect	Sum of Square (type III)	d.f.	Quadratic mean	F	**P**
Mean Racing Speed	Age	1.488	8	0.186	5.674	< 0.0001**
Best Time	Age	1.589	8	0.199	3.396	0.002**
Number of Races	Age	3,626.641	8	453.33	20.651	< 0.0001**
Number of Placements	Age	1,297.95	8	162.244	6.862	< 0.0001**

** *P* < 0.01. d.f.: degree of freedom; F represents statistic

## Discussion

The first aim of our research was to describe the prevalence of EP in IS racehorses, hypothesizing a high prevalence of this infection in our sample. Although many studies have been published regarding the epidemiology of EP within specific countries, their results are often contrasting due to variations in the experimental design, sample population, and diagnostic testing [4–6, 11–13]. It has been shown that exposure to vectors due to the geographic area, altitude, and grazing status, together with breed-dependent susceptibility, may correlate with the prevalence of the infection [14–16]. The present study included horses in coastal areas that were housed in boxes and did not graze; all examined horses were negative for *B. caballi*, and the prevalence of PCR-positivity for *T. equi* was 36.3%. Previously reported PCR positivity rates in southern Italy ranging from 38.5 to 81.4% for *T. equi* and from 0 to 17.6% for *B. caballi*. Such percentages were obtained testing only seropositive horses belonging to quite different geographical areas [4]. Nonetheless, considering influence of the soil, climatic zone and province location, our results can be considered quite similar (36.3% vs. 38.5%) [4, 5].

Even if clinically healthy racehorses with an excellent immune status were tested in this study, considering that protection against EP involves both innate and adaptive immunity [7, 10], one out of three tested horses were PCR-positive for *T. equi.* Age and sex affected the prevalence of EP in the present study. In the literature, there is no agreement about the effect of these variables on the occurrence of the infection. Gender was not significantly associated to the prevalence of disease in a study including 3100 horses, with no clinical signs of piroplasmosis [17]. In contrast with the results of Bartolomé Del Pino and *coll.* [4], our mares appeared to have a higher level of resistance to *T. equi* infection. On the other hand, a significantly higher seropositivity for *B. caballi* but not for *T. equi* has been previously reported in males [18]. Similarly, stallions were overrepresented in the PCR-positive – ELISA positive group, compared to PCR-negative – Elisa positive and control (PCR-negative – Elisa-negative) group [19]. The different grazing statuses of the tested horses may explain the contrasting results. A significantly higher prevalence of PCR positivity in older horses is in contrast with the results of the study by Bartolomé del Pino and *coll.* [4], who found that the odds ratio decreased with increasing age, but in accordance with the cumulative age-dependent increase in prevalence reported by Rüegg and *coll.* [20] and Montes Cortés and *coll*. 2017 [17]. The different inclusion criteria applied in the studies may explain the different results because age classes were nonhomogeneous between the studies. Horses were divided into 3 age classes (< 6, 6–12, and > 12 years) by Bartolomé del Pino and *coll.* [4], whereas they were aged between 3 and 11 years in the present study. The factors influencing the presence of *T. equi* in the blood across the life of a horse need further validation. Exposure to the infective agent should increase with increasing age; on the other hand, PCR-positivity could decrease due to parasite clearance that occurs at approximately 4 years [19, 21].

The second hypothesis we issued was that no differences in haematological and biochemical values could be detected between clinically healthy PCR-positive and PCR-negative horses. Few of them actually differed. Specifically, RBC count, Hgb, MCHC, AST, GGT, ALKP values were significantly higher in PCR-negative horses; BUN and Alb/Glob were higher in PCR-positive horses, although the mean values were within the normal physiological ranges, and values above the normal ranges were equally represented in both groups. In a recent study, MCV and MCH were higher in PCR-negative – ELISA positive compared to both PCR-positive and control groups [19], but not MCHC as in our sample. Moreover, it was not found any significant difference in RBC count or Hgb probably due to a higher number of stallions in PCR-negative – ELISA positive and PCR-positive groups, known that intact male horses bear a higher number of RBC and Hgb [19]. Differences in biochemical parameters are conflicting compared to other studies [19]. However, since recorded values fell within the physiological range in our and other studies [19], they are probably not worth noting.

Hence, the absence of obvious changes in haematological and biochemical parameters, compared to both the physiological range and PCR-negative horses, highlights the need for specific diagnostic tests, such as immunofluorescent antibody tests (IFATs) and PCR, to identify chronically infected horses [5, 6, 9, 11, 12, 17, 22]. Indeed, it has been recently demonstrated that the IFAT displays the best performance in assessing the positivity for both parasites, whereas other tests commonly used are not as good [23]. Furthermore, it suggests that, if present, poor performance in clinically healthy, PCR-positive horses should not be exclusively attributed to blood chemistry and haematological changes. In contrast with the present results, a significant reduction in the TP level and increases in the levels of TBil, AST, GGT, CK and ALKP had been reported in racing horses [6], which, unlike the horses in the present study, were symptomatic and clinically affected by EP.

Haematological and biochemical parameters are considered physiological parameters and are assessed according to laboratory reference values; however, many factors, such as sex, age, breed, and pregnancy can affect the ranges of normal values [19, 24–26]. For all significantly different variables between groups and within groups, analyses to test the effects of signalment data were performed. Few parameters were significantly influenced by age and bodyweight. These results are in partial agreement with those in the literature, reported by studies with samples that were heterogeneous in terms of age, breed, and use [27–31]; our IS racehorses were within the reference ranges for bodyweight and age according to the competition rules. Variability in biochemical parameters was evaluated to exclude bias due to differences in signalment data between PCR-positive and PCR-negative horses, which resulted older than PCR-positive. Indeed, differences in RBC count, Hgb, BUN, AST and Alb/Glob values were affected exclusively by PCR positivity/negativity, whereas the negative correlation between age and the AlkP concentration, detected in only the PCR-positive group, could be related to the younger age of the positive horses [32, 33].

Moreover, a difference in performance between PCR-positive and PCR-negative IS racehorses was not detected. There are many individual occasional or environment-related variables that can affect performance [29], and the evaluation of a single parameter can introduce bias [34]. Therefore, many criteria for racing performance have been included in this study, as previously suggested [35, 36]. Nonetheless, the correlation and PCA analysis finally suggested to evaluate only the best racing time among the performance metrics included. Even if earlier analyses reported that stallions have better racing performance than both mares and geldings [37–39] in this study, sex did not influence performance and, in agreement with previous reports [38, 39], improved with age. Differences in management, training, and breeding techniques, as well as in the inclusion criteria, may explain these contrasting results. Even if not statistically evaluated, management, feeding and training practices were considered superimposable between the training yards included. In agreement with the literature, our results suggest that the mean racing speed, number of placements and best time increased and improved, respectively, with age in both the PCR-negative and PCR-positive groups. The strength of this lack of difference between the two groups might be emphasized by the lack of difference in the number of races ran. It has been shown in German Trotters that younger animals have slower finish times [38, 40]. Similarly, the evaluation of a sample of North Swedish Trotters, aged between 3 and 12 years, showed that younger horses had slower best times. Moreover, older animals (7 to 12 years old) had a higher percentage of race wins than younger animals (3 to 6 years old) [39]. Finally, in our sample, PCR-positive and PCR-negative clinically healthy horses did not differ in terms of the performance metrics, i.e., the best racing time. This may indicate that chronic infection in well-managed and trained, clinically healthy horses does not imply a change in performance.

Muscle atrophy is a symptom of various equine diseases, and it has been suggested that even minor muscle loss may negatively affect athletic performance in horses [11, 41, 42]. Only a moderate or a mild degree of muscle atrophy was found in the present sample population, which consisted exclusively of well-trained horses. However, the MMS was correlated with management only in PCR-negative horses, probably implying an effect of management on muscle development that disappeared in PCR-positive horses. On the other hand, the MMS of PCR-positive horses was correlated with performance parameters, i.e., a moderate degree of muscle atrophy was associated with a decrease in performance compared to the lack of atrophy. The presence of an autoimmune inflammatory myopathy, with the upregulation of inflammatory cytokines that can cause myofiber atrophy and degeneration, has been demonstrated in muscle biopsies of horses with chronic EP [9]. Hence, we hypothesize that a subclinical myopathy might be the cause underlying such subtle changes in muscle mass. While severe muscle loss is easily detected, minor progressive changes require routine monitoring, and a clinical score system may be of great value [40].

A limitation of the present study was that muscle mass was evaluated subjectively by using a clinical score, and muscle biopsies were not performed because they are considered an invasive diagnostic method that is not appropriate for the evaluation of clinically healthy PCR-positive racehorses. However, the clinical MMS has been validated in companion animals, with substantial intrarater agreement [43]. Furthermore, the MMS has been previously used in horses to evaluate the effect of nutritional supplementation [44]. A highly detailed muscle scoring system to evaluate muscle development in dressage horses was proposed by Walker and *coll.* [45]. A simpler muscle atrophy scoring system (MASS) was recently validated in healthy and ill horses by Herbst and *coll.*, with the specific goal of evaluating muscle development and atrophy in the neck, back and hind regions [46]. The MMS we applied, on the other hand, included all the visually explorable and palpable major muscle groups. It might hypothesize that muscular atrophy in the PCR-positive group is linked to the EP myositis, but the absence of muscular histopathology prevents us from further considerations.

## Conclusion

The occurrence of PCR positivity for *T. equi* in IS racehorses is higher than 30%. Many factors can influence the chance that sport horses encounter vector insects; however, the clinical manifestation of the disease appears to be contained by the good management of racehorses. The performance metrics, haematological parameters and serum biochemistry values were not significantly different between PCR-positive and PCR-negative IS racehorses. Hence, the identification of positive horses requires specific diagnostic tests. A low MMS in PCR-positive horses was detected, but it was not further evaluated by histopathology. The use of muscle biopsies in the diagnosis of positive horses could be suggested for the early identification of autoimmune myositis, even in the absence of other clinical signs.

## Methods

All procedures were carried out as part of routine clinical evaluations and with the owner’s written informed consent in accordance with the Guiding Principles in the Care and Use of Animals approved by Italian law, and all procedures were approved by the Ethical Animal Care and Use Committee of the University of Napoli Federico II (protocol number 2019/0032059). This manuscript was prepared following the strobe guidelines and checklist [47].

This cross-sectional study was performed over a two-years period on the premises of seven yards in the Campania region (southern Italy), where the animals were regularly housed. The study included horses of both sexes, fully trained and ready to compete. Horses that had been diagnosed with orthopaedic, respiratory, or cardiovascular diseases in the last six months or at the time of the clinical examination were excluded. Horses that experienced a recent, i.e., less than six months, change in trainer/driver or owner were also excluded.

A form was completed for each yard and each horse, noting the signalment, anamnestic and management data of the horse. The body weight in kilograms was assessed using the heart girth and body length measurements in centimetres [48].

The presence/absence of muscle atrophy was scored by assigning a subjective muscle mass score (MMS) from 0 to 3, based on definition and mass of all visible and palpable major muscle groups, i.e., the neck, pectoral, shoulder and anconeal, gluteal, back and thigh muscles. A value of 0 indicated the absence of atrophy, 1 indicated mild atrophy, 2 indicated moderate atrophy and 3 indicated severe atrophy. The evaluation was always subjectively performed by the same operator (M.P.P., former Professor in veterinary surgery, with an experience as an equine practitioner of > 25 years) [44, 46].

### Blood collection

Jugular blood samples were obtained by qualified veterinarians between 7:00 and 8:00 a.m., before the morning feeding and the daily training after resting the previous day. Blood samples were collected into two vacuum tubes (Vacutainers; Becton Dickinson, Franklin Lakes, NJ) for serum biochemistry and two tubes with EDTA for a CBC and real-time PCR for *T. equi* and *B. caballi*. The samples for the CBC and serum biochemistry tests were sent to the laboratory on ice packs within two hours of collection, immediately centrifuged and processed. Cooled samples for real-time PCR were sent to the Istituto Zooprofilattico Sperimentale Lazio e Toscana (IZSLT - Rome, Italy) laboratory within 36 h of collection.

### Molecular tests

Real-time PCR was performed following the protocols described by Bartolomè Del Pino and *coll.* for *T. equi* and *B. caballi* [4].

*DNA extraction* – DNA extraction was performed using the automated robotic workstation QIAcube HT (Qiagen, GmbH, Hilden, Germany) and the QIAamp cador Pathogen Mini kit (Qiagen) according to the manufacturer’s instructions. The DNA was eluted in 60 µl of AVE buffer included in the kit and stored at -80 °C.

*Real-time PCR for B. caballi and T. equi* – The *T. equi* rtPCR protocol employed was that reported by Kim and *coll.* [49], amplifying an 81-bp fragment 108 outside the V4 hypervariable region of the 18 S rRNA gene, while that for B. caballi was performed according to Bhoora and *coll.* [50], which amplifies a 95-bp fragment within the V4 hypervariable region 110 of the 18 S rRNA gene. The TaqMan ® Universal PCR Master Mix kit (A. Biosystems, Foster City, CA, 111 USA) was used for both real-time PCRs.

### Haematology and serum biochemistry

The CBC was performed using a cell counter (HeCo Vet 5; Seac, Firenze, Italy), and a complete biochemical profile (blood urea nitrogen [BUN], creatinine [CREA], total bilirubin [Tbil], BUN-to-creatinine ratio [BUN/CREA], glucose [Glu], ionized calcium [Ca], aspartate aminotransferase [AST], alkaline phosphatase [ALKP], lactate dehydrogenase [LDH], creatine kinase [CK], gamma-glutamyl transferase [GGT], total protein [TP], albumin [Alb], Alb-globulin [Glob] ratio, Glob) was determined using a Catalyst Dx™ Chemistry Analyzer (IDEXX, Westbrook, Maine).

### Performance evaluation

The data from the 30 races preceding the blood sampling were obtained from the official racing website Ippica Biz (by IPPICA E STAMPA srl, http://www.ippicabiz.it). The number of total and placed races, the number of victories, mean racing speed, the best racing time and total earnings were recorded [34, 36, 51–54]. The mean racing speed was the average speed maintained by the horse over the last 1000 m of a race (m/s).

### Statistical analysis

All data were recorded on electronic spreadsheet (Excel© Microsoft for Mac, v. 16.43, Redmond, WA, USA) and then imported into statistical software (Prism©, GraphPad Software, Inc., v. 8.2.0, San Diego, CA, USA; IBM® SPSS® Statistics, IBM Corp., v. 26.0.0.0, Armonk, NY, USA; JMP Pro, v. 16.0, SAS Institute, Cary, NC, USA). The sample was divided into two groups, defined as Positive and Negative based on the PCR results. The normality of the distribution of the data was tested with the Shapiro-Wilk *W* test. Normally distributed data are reported as means ± standard deviations, and non-normally distributed data are reported as medians (ranges).

Power calculation for the detection of differences between two independent means was performed using G*power^®^ 3.1 software; the tests were two-tailed, with an effect size *d* of 0.5 [55]. A calculation for the optimal sample size was performed.

The possible association of PCR-positivity with sex was evaluated with Fisher’s exact test, and the odds ratio (O.R.) and relative risk (R.R.) and their respective 95% confidence intervals (95%CI) were calculated.

Differences in CBCs and biochemical parameters between positive and negative horses were analysed. Comparisons were performed with pooled Student’s *t* tests, and *F* tests were used to compare variances for normally distributed data (bodyweight, red blood cell [RBC] counts, haemoglobin [Hgb] concentrations, mean corpuscular haemoglobin concentration [MCHCs], white blood cell [WBC] counts). When variances were significantly different, Welch’s correction was applied (haematocrit [Hct], platelets [PLTs], alkaline phosphatase [AlkP]). The Mann-Whitney *U* test was used for non-parametric data (age, mean corpuscular volume [MCV], MCHC, red cell distribution width [RDW], mean platelet volume [MPV], procalcitonin [Pct], platelet distribution width [PDW], lymphocytes [Lym], neutrophils [Neu], monocytes [Mon], eosinophils [Eos], basophils [Bas], BUN, TBil, CREA, BUN/CREA, Glu, TP, Alb, Glob, Alb/Glob, Ca, AST, GGT, CK, LDH). For all variables that were significantly different between the two groups, an intragroup univariate general linear model was applied to evaluate if signalment data (age, sex, or bodyweight) had any effect. Within-group correlations were tested with Spearman’s rank correlation (r_s_) (bodyweight vs. MCHC POS; age vs. Glob NEG).

Contingency tables and chi-square tests were used to explore potential associations of PCR positivity and training yard with the MMS; the same approach was repeated to evaluate the potential association between the training yard and the MMS within each group.

Performance metrics were tested by a correlation matrix, principal component analysis (PCA) for covariance, scree plot evaluation and subsequently orthogonal rotation with Varimax algorithm for the number of variables suggested by the scree plot itself. Then, the effects of PCR positivity, MMS and training yard on the performance metrics selected were tested by a mixed model, with each performance metric as a dependent variable, PCR positivity and MMS as fixed effects, and the training yard as a random effect. A univariate general linear model with the MMS as a fixed effect, the training yard and MMS within the training yard group as random effects, and the selected performance metrics as the dependent variable was constructed to evaluate within-group effects on the aforementioned performance metrics. Bonferroni correction for multiple comparisons was performed *post hoc* between the different MMS score groups. The total number of races was compared between groups to exclude differences, which would have led to the exclusion of number of placed races and number of victories from further analysis.

Regardless the PCA results on performance metrics, signalment data were evaluated for any effect on each of them with a univariate general linear model. *Post hoc*, Student’s *t* or the Mann-Whitney’s *U* test or Spearman’s rank correlation (r_s_) test were used, depending on type – continuous or categorical – and according to the data distribution. A *P* value < 0.05 was considered significant for all tests.

## Data Availability

Raw data are available from the corresponding author upon reasonable request.

## Abbreviations

EP: Equine piroplasmosis
IS: Italian Standardbred
PCR: Polymerase chain reaction
O.R.: Odds ratio
R.R.: Relative risk
95%CI: 95% confidence interval
CBC: Complete blood count
RBC: Red blood cells
Hgb: Hemoglobin
MCHC: Mean cell hemoglobin concentration
Hct: Hematocrit
MCV: Mean cell volume
MCH: Mean corpuscular hemoglobin
RDW: Red cell distribution width
Plt: Platelets
Pct: Plateletcrit
PDW: Platelet distribution width
WBC: White blood cells
Lym: Lymphocytes
Neu: Neutrophils
Mon: Monocytes
Eos: Eosinophils
Bas: Basophils
Glob: Globulin
Alb/Glob ratio: Albumin to globulin ratio
BUN: Blood urea nitrogen
AST: Aspartate amino-transferase
AlkP: Alkaline phosphatase
GGT: Gamma glutamyl-transferase
TBil: Total bilirubin
Crea: Creatinine
Glu: Glycemia
TP: Total proteins
Alb: Albumin
Ca: Calcium
CK: Creatin-kinase
LDH: Lactate dehydrogenase
MMS: Muscle mass score
ELISA: Enzyme-linked immunosorbent assay
IFAT: Immunofluorescent antibody test
MASS: Muscle atrophy scoring system
